# Impact of COVID‐19 and factors associated with long COVID and COVID‐19 vaccine uptake in people with HIV in the United Kingdom: Results from Positive Voices 2022

**DOI:** 10.1111/hiv.70026

**Published:** 2025-05-01

**Authors:** F. Nakagawa, R. Palich, M. Kall, J. Sewell, C. Smith, C. Kelly, H. Kitt, A. Pelchen‐Matthews, A. Aghaizu, A. Sparrowhawk, N. Mackie, T. Djuretic, S. Schoeman, C. Humphreys, M. Lipman, F. C. Lampe, A. J. Rodger, the PV2022 Study Group, Ann Sullivan, Rachel Jones, Mohammed Hassan, Serge Miodragovic, Ann Sullivan, Victoria Tittle, Mohammed Hassan, Serge Miodragovic, Sarah Edwards, Melanie Rosenvinge, Allison Mascagni, Rosa Harrington, Claudia Adade, Emily Clarke, Sandra Mason, Emily Clarke, Elaine Priest, Emily Clarke, Sandra Mason, Emily Clarke, Elaine Priest, Emily Clarke, Melissa Martin, Athavan Umaipalan, Julie Field, Clare Woodward, Felicity Williams, Liz Hamlyn, Lucy Campbell, Steve Taylor, Gerry Gilleran, Satwant Kaur, Cathy Ormiston, Laura Wilson‐Powell, Kate Saunders, Meena Gupta, Julia Ball, Mark Gompels, Louise Jennings, Malgorzata Slowinska, Angela Bailey, Sandra Rushwaya, Sarah Schoeman, Tadas Mazeika, Shalini Andrews, Laura Noonan, Liz Hamlyn, Lucy Campbell, Darren Cousins, Catherine Oliver, Daniel Clutterbuck, Connor Dalby, Amy Shepherd, Anura Piyadigamage, John Martin, Judith Zhou, Barbara Hayman, Emma Rutland, Paola Cicconi, Charlie Wells, Fiona Wilson, Claudia Krause, Su Jenkins, Anitha Vidhyadharan, Samantha Harwood, Lisa Goodall, Alison Bridgwood, Laura Wilson‐Powell, Suzanne Francis, Kirsty Mynard, Mandy Austin, Judith Zhou, Farai Mukazi, Chloe Hoskins, Ian Cormack, Jonathan Shaw, Amanda Smith, Nadia Khatib, Julie Walsh, Sarah Schoeman, Tadas Mazeika, Anitha Vidhyadharan, Brenda Hollier, Martin Jones, Penny Boxall, Ade Apoola, Catherine Gatford, John Evans‐Jones, Jennifer Harrison, Nashaba Matin, Moses Shongwe, Nashaba Matin, Moses Shongwe, Ian Fairley, Martin Jones, Zoe Cuthbertson, Penny Boxall, Chitra Babu, Denise Donahue, Fiona Burns, Katie Spears, Thomas Fernandez, Graham McKinnon, Rachael Bridgman, Ann Sullivan, James Hardie, Mohammed Hassan, Serge Miodragovic, Amanda Clarke, Lisa Barbour, Carole Cable, Mohanarathi Kawsar, Memory Kakowa, Richard Gilson, Gosala Gopalakrishnan, Abigail Severn, Anitha Vidhyadharan, Kate Castro‐Sanchez, Andrew Ustainowski, Fahd Niaz, Sophie Herbert, Helen Reboul, Ashini Fox, Sarah Chadwick, Nelson David, Megan Khan, Andrea Ng, Julia Rogers, Katie Saunders, Andrea Ng, Julia Rogers, Katie Saunders, Cathy Ormiston, Amandeep Gill, Laura Wilson‐Powell, Katie Saunders, Alison Blume, Natalie Parker, Jonathan Ross, Sindiso Masuka, Olubanke Davies, Analyn Alipustain, Maheshraj Radhakrishnan, Nadi Gupta, Nicola Williams, Helen Iveson, Ian Fairley, Angela Bailey, Sandra Rushwaya, Emma Street, Andrew Sealy, Ian Fairley, Tom Yucebiyik, Elbushra Herieka, Kevin Turner, Alison Blume, Felicity Young, Karen Rogstad, Jessica Mcneill, Gareth Stephens, Zoe Warwick, Angela Robinson, Elaine Freeman, Nashaba Matin, Moses Shongwe, Laura Hilton, Donna Stookes, Sarah Schoeman, Tadas Mazeika, Elizabeth Okecha, Nicola Fearnley, Jackie Todd, Sue Kimachia, Ann Sullivan, Sarah Edwards, Mohammed Hassan, Sophie Herbert, Helen Reboul, Jessica Daniel, Mary‐Jane Harding, Jonathan Shaw, Abbey Eboigbe, Ashley Hanson, Liz Hamlyn, Katie Toler, Fabian Chen, Emma Wainwright, Felix Kpodo, Nisha Pal, Clare Megson, Anitha Vidhyadharan, Matt Waller, David Chadwick, Jessica Roberts, Iain Reeves, Tracey Fong, Raouf Moussa, Melissa Milsom, Emma Street, Mike Ward, Lucy Twigger, Charlotte Swift, Raj Patel, Jane Whitehead, Sathish Thomas William, Jane Holder, Stephen Kegg, Rosa Harrington, Allison Mascagni, Claudia Adade, Lewis Haddow, Jessica Osorio, Ann Sullivan, Marie‐Louise Svensson, Sandra Underwood, Helen Pollitt, Anitha Vidhyadharan, Brenda Hollier, Ann Sullivan, Samantha Hill, Sara Scofield, Jenny Murira, Nicola Mackie, Sophia Taylor, Romina Tajik, Orla McQuillan, Denise Donahue, Judith Zhou, Rebecca Murdock, Elaine Banks

**Affiliations:** ^1^ Institute for Global Health, University College London London UK; ^2^ UK Health Security Agency London UK; ^3^ George House Trust Manchester UK; ^4^ Imperial College Healthcare NHS Trust London UK; ^5^ Brotherton Wing Clinic, Leeds General Infirmary Leeds UK; ^6^ Respiratory Medicine University College London London UK; ^7^ Royal Free London NHS Foundation Trust London UK

**Keywords:** COVID‐19, HIV, long COVID, vaccination

## Abstract

**Objectives:**

We assessed the impact of COVID‐19, and the prevalence and factors associated with a history of COVID‐19 infection, long COVID and incomplete COVID‐19 vaccine uptake among people with HIV.

**Methods:**

Positive Voices 2022 is a questionnaire study of people accessing HIV care in the United Kingdom (March 2022–April 2023). Logistic regression assessed factors associated with a history of COVID‐19 (previous positive test), long COVID among those with a history of COVID‐19 (ongoing symptoms, with COVID‐19 onset >3 months previously) and incomplete COVID‐19 vaccine uptake (less than three doses of vaccine), adjusted for: age; gender; ethnicity; and year of HIV diagnosis.

**Results:**

In all, 4188 participants were included. Commonly reported negative impacts of the pandemic were on social contact (44% of participants), mental health (30%), healthcare access (26%) and financial security (25%). Overall, 2068 of 4188 (49.4%) participants had a history of COVID‐19. Of these, 10.8% met criteria for long COVID, associated with female gender, unemployment, financial hardship, earlier HIV diagnosis date, diabetes diagnosis, asthma/chronic obstructive pulmonary disease diagnosis, obesity and symptoms of depression and anxiety. Overall, 95.8% reported having at least one vaccine dose, but 649 (15.7%) participants had incomplete vaccine uptake, associated with younger age, female gender, Black African ethnicity, lower education, financial hardship, unemployment, multioccupancy household, more recent HIV diagnosis, detectable HIV viral load and symptoms of depression and anxiety.

**Conclusions:**

About half of participants had a history of COVID‐19, of whom 11% had persistent symptoms (long COVID). COVID‐19 vaccine uptake was high, but incomplete uptake was apparent for 16% of participants and was more common among women, younger people, Black African individuals and those with socio‐economic disadvantage.

## INTRODUCTION

The COVID‐19 pandemic was one of the most serious public health events of the 21st century, resulting in more than 250 000 deaths in the United Kingdom [1, 2]. From early in the pandemic, people with HIV were identified as a clinical risk group and prioritized for vaccination [3]. Evidence for an increased risk of COVID‐19 morbidity and mortality in people with HIV has been mixed. One systematic review and meta‐analysis, in which most studies were from the United States or Europe, found no association between HIV status and risk of death [4], whereas another using global data from hospitalized people found HIV to be an independent risk factor for severe COVID‐19 at admission and in‐hospital mortality [5].

Increased susceptibility to severe COVID‐19 in people with HIV may partly relate to risk factors that also affect the general population, such as being male, older age and having the presence of comorbid conditions (including diabetes, obesity, hypertension, and pulmonary, liver and cardiovascular diseases) [5, 6, 7]. HIV‐specific risk factors for more severe disease include having a current CD4+ T‐cell count <200 cells/mm^3^ [5, 8, 9, 10]. In people with HIV with CD4 T‐cell counts above this cut‐off, associated comorbidities and age were the most important predictors of COVID‐19 severity, although increased immune activation and persistent chronic inflammation may also play a role [9]. However, COVID‐19 and HIV both disproportionately affect people of minoritized ethnicity and those impacted by socio‐economic deprivation [11, 12, 13, 14]. In addition, disruption of healthcare provision had additional consequences for people with long‐term health conditions such as HIV, as it led to reduced face‐to‐face consultations, reduced blood monitoring and extended durations between appointments [15].

COVID‐19 vaccines are highly effective in preventing severe forms of disease and reducing mortality [16, 17]. The vaccines are safe, with no significant difference in side effects for people with HIV compared to the general population [18]. Individuals on virally suppressive antiretroviral therapy (ART) with good CD4 counts generate an effective immune response following vaccination, similar to people without HIV [19]. Immune response is less robust in people with low CD4 counts (<200/mm^3^) with evidence suggesting that a third primary dose of the COVID‐19 vaccine is effective in boosting a protective immune response against severe COVID‐19 disease in this group [19, 20]. In the United Kingdom, further booster doses were also recommended for all people with HIV [21].

Globally, COVID‐19 vaccine uptake among people with HIV has been varied. Several studies in people with HIV reported that factors such as race/ethnicity, gender and level of education, as well as mistrust of vaccination, were associated with COVID‐19 vaccine hesitancy, which aligns with what is seen in the general population [22, 23, 24, 25, 26].

Using data from the Positive Voices 2022 study, the largest UK national survey in people with HIV accessing outpatient care, we describe the reported impact of the COVID‐19 pandemic on daily life among people with HIV and investigate the prevalence and factors associated with: having a history of COVID‐19, having persisting symptoms post infection (long COVID) and incomplete COVID‐19 vaccination uptake.

## METHODS

### Study design, setting and participants

Positive Voices is a multi‐site, cross‐sectional questionnaire study conducted in adults living with HIV attending 101 outpatient NHS HIV specialist clinics in England, Wales and Scotland, carried out from April 2022 to March 2023. A random sample of people was selected for recruitment at each participating study site using the clinic's most recent year HIV and AIDS Reporting System (HARS) national UK surveillance return. From December 2022, 14 clinics in London adopted a sequential recruitment strategy to increase recruitment. All participants self‐completed a confidential questionnaire (both paper and online options were available) on demographic, socio‐economic, HIV‐related, health and lifestyle factors, health service needs and usage and sexual behaviour. The study was approved by the London Harrow Research Ethics Committee (ref. 13/LO/0279) and the methods have been published elsewhere [27].

### Impact of COVID‐19 on daily life

The survey asked about the impact of the pandemic on different areas of life. Participants were asked to indicate whether they had experienced any of the following because of COVID‐19: loss of employment/income; increased workload; having to work from home; housing insecurity; financial insecurity; food insecurity; difficulty accessing healthcare/medication; worse mental health; reduced access to support services; relationship/family problems; increased caring responsibilities; lack of social contact; bereavement.

### History of COVID‐19

Participants were asked if they had ever had COVID‐19, with possible response options: ‘Yes, I've had a positive test’; ‘I had symptoms but no positive test’; ‘I had symptoms but never tested’; ‘No’; ‘Don't know’. A history of COVID‐19 was considered positive only for those who indicated that they had a previous positive test.

### Post COVID‐19 syndrome (long COVID)

All participants who reported that they had previously had a positive COVID‐19 test were asked to give the date (month and year) when they first had COVID‐19 (either positive test result or onset of symptoms). They were then asked, ‘Are you continuing to experience symptoms due to your COVID infection?’. Post COVID‐19 syndrome (‘long COVID’) is defined as symptoms that develop during or after COVID‐19, continue for more than 12 weeks and are not explained by an alternative diagnosis [28, 29]. In this analysis, we considered participants had long COVID if they indicated the presence of ongoing symptoms due to COVID‐19 and the date reported for the first positive COVID‐19 test or onset of symptoms was at least 3 months prior to the questionnaire date.

### COVID‐19 vaccine uptake

Participants were asked how many COVID‐19 vaccine doses they had received with response options of zero to four vaccine doses. At the time the survey was conducted (2022–2023), the current Joint Committee on Vaccination and Immunisation (JCVI) and British HIV Association (BHIVA) guidance was for people with HIV to have received three or four doses depending on CD4 count, detectable HIV viraemia, recent serious HIV related illness and non‐receipt of ART [21]. Therefore, a complete course of vaccination was defined as having received three or more doses. Participants who had received zero, one or two doses were classified as having incomplete vaccination uptake. Participants who indicated they had not received all the recommended COVID‐19 vaccine doses were asked to state reasons for not doing so by ticking all that applied out of the following options: ‘Side effects after one or more of the doses’; ‘Not been offered one or more of the recommended doses’; ‘Cannot receive all recommended doses for medical reasons’; ‘Planning to receive all recommended doses but have not done so yet’; ‘Do not want to have all recommended doses’; ‘I do not wish to be vaccinated’; and final option of ‘Other’ with the option of free text to provide further detail.

### Demographic, socio‐economic, HIV‐related, health and lifestyle factors

Other factors considered in analyses included: age group (18–34, 35–44, 45–54, 55–64, 65+ years), gender (male, female, non‐binary and other, undisclosed), ethnicity (White, Black African, Black other, Asian, mixed, other including undisclosed), region (London, Midlands and East, North, South, Scotland/Wales/unknown), highest level of education (to age 16, to age 18 or post‐16 qualification, undergraduate degree, postgraduate degree, other/missing), employment status (employed, unemployed, retired, not working due to sickness or disability, other including student and carer), having enough money to meet basic needs (yes always, most of the time, some of the time, no), household composition (living alone, 2 people living in household, 3–5 people, >6 people), year of HIV diagnosis (1995 or earlier, 1996–2001, 2002–2007, 2008–2013, 2014–2023), self‐reported viral load (undetectable, detectable, unknown/missing), ever‐diagnosed with cardiovascular disease, diabetes, asthma or chronic obstructive pulmonary disease (COPD), self‐reported body mass index based on self‐reported height and weight (BMI; ≥30 kg/m^2^, <30 kg/m^2^) and number of sexual partners in the last 3 months (0, 1, 2–5, >6 people). Symptoms of depression and anxiety were assessed using PHQ‐9 and GAD scores respectively using a definition of total score >=10 in each case. We also defined a variable ‘demographic group’ based on gender, sexuality and ethnicity, with seven categories as follows: GBMSM (gay, bisexual and other men‐who‐have‐sex‐with‐men), Black African heterosexual men, other heterosexual men, Black African women, other women, non‐binary or other gender or undisclosed gender or sexuality.

### Statistical analysis

Characteristics were summarized for the study population. Associations of demographic, socio‐economic, HIV‐related, health and lifestyle characteristics with: (i) COVID‐19 history, (ii) ongoing symptoms consistent with long COVID among those with a history of COVID‐19 and (iii) incomplete vaccine uptake, were assessed using logistic regression, with unadjusted and adjusted odds ratios (aORs) with 95% confidence intervals (95% CI). In multivariable models, each factor of interest was included in a separate model adjusted for four pre‐defined ‘core variables’ only: age group, gender, ethnicity and year of HIV diagnosis group. All statistical analyses were performed in STATA version 18.0 (Stata Corp, College Station, TX, USA).

## RESULTS

In all, 4271 of 4622 (92.4%) survey participants answered at least one question on COVID‐19. We excluded a further 83 participants due to missing age or year of HIV diagnosis, resulting in a final sample size for this analysis of 4188, who are described in Table 1. Of these people, there were 2486 (59.4%) GBMSM, 251 (6.0%) Black African heterosexual men, 351 (8.4%) other heterosexual men, 555 (13.3%) Black African women, 445 (10.6%) other women, 28 (0.7%) people with non‐binary or other gender, and 72 (1.7%) with undisclosed gender or sexuality. Overall, 2768 (66.1%) people were of White ethnicity, 977 (23.3%) of Black ethnicity and 443 (10.6%) of other or undisclosed ethnicity. The median age of participants was 53 years (inter‐quartile range, IQR 44–60). Nearly all (4146; 99.0%) participants were currently taking ART with a median duration of 12 years (IQR 8–18) and 3893 (93.0% of all participants) self‐reported a suppressed viral load. Just under half of participants reported some level of financial insecurity: 1888 (45.1%) did not always have enough money to meet basic needs, and 276 (6.6%) never had enough money. One in 5 (843/4107 20.5%) participants had depressive symptoms (PHQ‐9 score ≥ 10) and 610/4105 (14.9%) had anxiety symptoms (GAD‐7 score ≥ 10).

**TABLE 1:** Characteristics of PV2022 survey participants and associations with a history of COVID‐19 infection.

		History of COVID‐19 infection (reported positive test) (*N* = 2068/4188)					
*N* = 4188^a^	*N* (column %)	*N*	%	Unadjusted odds ratio (95% CI)	*p*‐value	Adjusted odds ratio^c^ (95% CI)	*p*‐value
Age group (years)							
18–34	316 (7.5%)	201	63.6	1.22 (0.93–1.60)	<0.0001^b^	1.21 (0.92–1.60)	<0.0001^b^
35–44	805 (19.2%)	474	58.9	1		1	
45–54	1236 (29.5%)	612	49.5	0.69 (0.57–0.82)		0.71 (0.59–0.86)	
55–64	1284 (30.7%)	587	45.7	0.59 (0.49–0.70)		0.58 (0.48–0.71)	
65+	547 (13.1%)	194	35.5	0.38 (0.31–0.48)		0.36 (0.29–0.46)	
Combined demographic group^c^							
GBMSM	2.486 (59.4%)	1324	53.3	1	<0.0001	1	<0.0001
Black African heterosexual men	251 (6.0%)	102	40.6	0.60 (0.46–0.78)		0.62 (0.48–0.82)	
Other heterosexual men	351 (8.4%)	152	43.3	0.67 (0.54–0.84)		0.77 (0.61–0.97)	
Black African women	555 (13.3%)	238	42.9	0.66 (0.55–0.79)		0.63 (0.52–0.77)	
Other women	445 (10.6%)	216	48.5	0.83 (0.67–1.01)		0.81 (0.66–0.99)	
Non‐binary or other gender	28 (0.7%)	10	35.7	0.49 (0.22–1.06)		0.40 (0.18–0.87)	
Undisclosed gender or sexuality	72 (1.7%)	26	36.1	0.50 (0.30–0.81)		0.50 (0.31–0.82)	
Ethnicity							
White	2768 (66.1%)	1427	51.6	1	<0.0001	1	<0.0001
Black African	861 (20.6%)	359	41.7	0.67 (0.58–0.78)		0.65 (0.54–0.78)	
Black other	116 (2.8%)	44	37.9	0.57 (0.39–0.84)		0.56 (0.38–0.82)	
Asian	163 (3.9%)	84	51.5	1.00 (0.73–1.37)		0.92 (0.66–1.27)	
Mixed	125 (3.0%)	62	49.6	0.92 (0.65–1.32)		0.85 (0.59–1.22)	
Other including undisclosed	155 (3.7%)	92	59.4	1.37 (0.99–1.91)		1.21 (0.86–1.70)	
Gender							
Male	3133 (74.8%)	1596	50.9	1	0.0012	1	0.034
Female	1000 (23.9%)	454	45.4	0.80 (0.69–0.92)		0.94 (0.79–1.11)	
Non‐binary or other gender	28 (0.7%)	10	35.7	0.54 (0.25–1.16)		0.44 (0.20–0.96)	
Undisclosed	27 (0.6%)	8	29.6	0.41 (0.18–0.93)		0.41 (0.17–0.95)	
Region of HIV care							
London	1991 (47.5%)	1036	52.0	1	0.0093	1	0.065
Midlands and East	707 (16.9%)	329	46.5	0.80 (0.68–0.95)		0.86 (0.72–1.03)	
North	657 (15.7%)	311	47.3	0.83 (0.69–0.99)		0.82 (0.69–0.99)	
South	787 (18.8%)	365	46.4	0.80 (0.68–0.94)		0.81 (0.68–0.96)	
Scotland/Wales/unknown	46 (1.1%)	27	58.7	1.31 (0.72–2.37)		1.13 (0.61–2.07)	
Highest education level							
Education to age 16	983 (23.5%)	417	42.4	1	<0.0001	1	<0.0001
Education to age 18 or post‐16 qualification	1080 (25.8%)	508	47.0	1.21 (1.01–1.43)		1.18 (0.99–1.41)	
Undergraduate degree	944 (22.5%)	514	54.5	1.62 (1.36–1.94)		1.55 (1.29–1.86)	
Postgraduate degree	984 (23.5%)	542	55.1	1.66 (1.39–1.99)		1.60 (1.33–1.92)	
Other/missing	197 (4.7%)	87	44.2	1.07 (0.79–1.46)		1.17 (0.85–1.60)	
Employment status							
Employed	2729 (66.5%)	1535	56.2	1	<0.0001	1	<0.0001
Unemployed	308 (7.5%)	108	35.1	0.42 (0.33–0.54)		0.45 (0.35–0.58)	
Retired	569 (13.9%)	202	35.5	0.43 (0.35–0.52)		0.55 (0.43–0.71)	
Not working due to sickness/disability	367 (8.9%)	133	36.2	0.44 (0.35–0.55)		0.45 (0.36–0.57)	
Other including student, carer	129 (3.1%)	51	39.5	0.51 (0.35–0.73)		0.55 (0.38–0.80)	
Having money for basic needs (food, rent, bills etc.)							
Yes, always	2213 (54.0%)	1190	53.8	1	<0.0001^b^	1	<0.0001^b^
Most of the time	1073 (26.2%)	476	44.4	0.69 (0.59–0.79)		0.69 (0.59–0.80)	
Some of the time	539 (13.1%)	255	47.3	0.77 (0.64–0.93)		0.83 (0.68–1.02)	
No	276 (6.7%)	104	37.7	0.52 (0.40–0.67)		0.57 (0.43–0.74)	
Household composition							
Living alone	1438 (35.2%)	637	44.3	1	<0.0001^b^	1	<0.0001^b^
2 people	1731 (42.3%)	879	50.8	1.30 (1.13–1.49)		1.21 (1.05–1.40)	
3–5 people	859 (21.0%)	476	55.4	1.56 (1.32–1.85)		1.61 (1.34–1.95)	
6 people or more	63 (1.5%)	32	50.8	1.30 (0.78–2.15)		1.22 (0.72–2.05)	
Year of HIV diagnosis							
2014–2023	931 (22.2%)	508	54.6	1	<0.0001^b^	1	0.95^b^
2008–2013	1099 (26.2%)	570	51.9	0.90 (0.75–1.07)		1.02 (0.85–1.22)	
2002–2007	1100 (26.3%)	504	45.8	0.70 (0.59–0.84)		0.95 (0.79–1.15)	
1996–2001	610 (14.6%)	285	46.7	0.73 (0.59–0.90)		1.02 (0.82–1.27)	
1995 or earlier	448 (10.7%)	201	44.9	0.68 (0.54–0.85)		0.97 (0.76–1.25)	
Most recent viral load (self‐report)							
<50 copies/mL	3893 (93.0%)	1955	50.2	1	<0.0001	1	<0.0001
≥50 copies/mL	141 (3.4%)	54	38.3	0.62 (0.44–0.87)		0.65 (0.45–0.92)	
Don't know/missing	154 (3.7%)	59	38.3	0.62 (0.44–0.86)		0.65 (0.46–0.92)	
Cardiovascular disease							
No	3430 (90.6%)	1781	51.9	1	<0.0001	1	<0.0001
Ever‐diagnosed	356 (9.4%)	129	36.2	0.53 (0.42–0.66)		0.64 (0.50–0.81)	
Diabetes							
No	3412 (90.7%)	1740	51.0	1		1	
Ever‐diagnosed	349 (9.3%)	163	46.7	0.84 (0.68–1.05)	0.13	1.14 (0.90–1.43)	0.28
Asthma or COPD							
No	3146 (81.5%)	1608	51.1	1	0.11	1	0.029
Ever‐diagnosed	712 (18.5%)	340	47.8	0.87 (0.74–1.03)		0.83 (0.70–0.98)	
BMI (self‐reported)							
<30 kg/m^2^	2849 (76.0%)	1472	51.7	1		1	
Obese (≥30 kg/m^2^)	900 (24.0%)	425	47.2	0.84 (0.72–0.97)	0.020	0.96 (0.81–1.12)	0.58
Depressive symptoms							
No	3264 (79.5%)	1657	50.8	1	0.005	1	<0.0001
Yes (PHQ‐9 ≥10)	843 (20.5%)	382	45.3	0.80 (0.69–0.94)		0.73 (0.62–0.85)	
Anxiety symptoms							
No	3495 (85.1%)	1763	50.4	1	0.027	1	0.001
Yes (GAD‐7 ≥10)	610 (14.9%)	278	45.6	0.82 (0.69–0.98)		0.73 (0.61–0.87)	
Number of sexual partners in the last 3 months							
0	1261 (35.6%)	539	42.7	1	<0.0001^b^	1	<0.0001^b^
1	1316 (37.2%)	697	53.0	1.51 (1.29–1.76)		1.31 (1.11–1.54)	
2–5	583 (16.5%)	336	57.6	1.82 (1.49–2.22)		1.53 (1.24–1.88)	
>6	379 (10.7%)	244	64.4	2.42 (1.91–3.07)		1.96 (1.53–2.51)	

*Note*: Region: Midlands and East of England (East Midlands, East of England, West Midlands); North of England (North East, North West, Yorkshire and Humber); South of England (South East, South West). Column percentages may not add to 100% because of rounding. Missing categories if not incorporated into one of the above categories are included in analyses but not shown in the table.

Abbreviations: BMI, body mass index; COPD, chronic obstructive pulmonary disease; GBMSM, gay, bisexual and other men‐who‐have‐sex‐with‐men.

^a^ Number of missing values not included in categories specified in table: employment status (*n* = 86), money for basic needs (*n* = 87), household composition (*n* = 97), cardiovascular disease (*n* = 402), diabetes (*n* = 427), asthma or COPD (*n* = 330), self‐reported BMI/obesity (*n* = 439), depressive symptoms (*n* = 81), anxiety symptoms (*n* = 83), number of sexual partners in the last 3 months (*n* = 649).

^b^ *p*‐value refers to test for trend.

^c^ Every variable was considered in a separate model for all results. Multivariable models adjusted for age group, ethnicity, gender and year of HIV diagnosis.

### Impact of the pandemic on daily life

**FIGURE 1:**
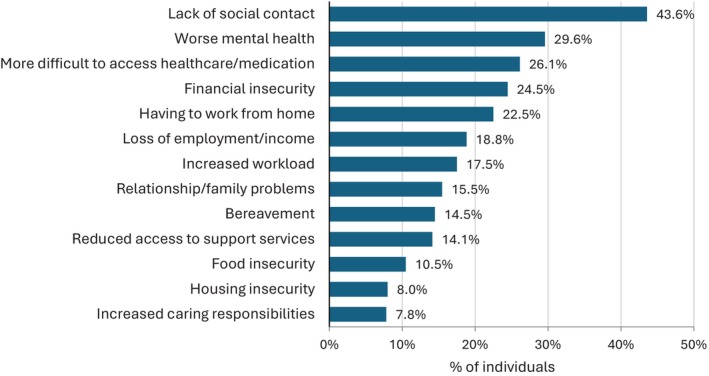
Impact of COVID‐19 pandemic on multiple areas of daily life (*n* = 4188).

The most frequently reported impact of the COVID pandemic was lack of social contact (1825/4188, 43.6%), followed by worse mental health (1238/4188 29.6%), difficulties accessing healthcare/medication (1094/4188 26.1%), financial insecurity (1027/4188 24.5%) and having to work from home (942/4188 22.5%). Factors reported by 15% to 20% of participants were loss of employment/income, increased workload and relationship/family problems. (Figure 1). Between 10% and 15% of participants reported bereavement, reduced access to support services, and food insecurity.

### History of COVID‐19 and associated factors

In all, 2068 of 4188 (49.4%; 95% CI: 47.9%‐50.9%) reported a previous positive COVID‐19 test. In adjusted analysis, a COVID‐19 history was more frequent among younger participants, GBMSM compared to all other demographic groups, people with a higher level of education, people in employment, people who always had money to meet basic needs, people who lived in a household of two or more compared to living alone, people with self‐reported undetectable viral load compared to detectable or unknown and those with a higher number of sexual partners in the last 3 months. A COVID‐19 history was less common among people of Black ethnicity compared to White or other ethnicities, people whose gender was non‐binary/other or undisclosed compared to men and women, among those diagnosed with a cardiovascular disease, asthma or COPD and among those with symptoms of depression or anxiety (Table 1).

### Post COVID‐19 syndrome (long COVID) among those with a history of COVID‐19

Out of the 2068 participants who indicated previously testing positive for COVID‐19, 1844 gave a reliable date of infection (between 2020 and date of survey completion) and had also answered questions regarding ongoing COVID‐19 symptoms. Among this group with a history of COVID‐19, 200/1844 (10.8%), 95% CI (9.5%–12.4%) reported they were continuing to experience symptoms at the time of survey completion and met the definition of long COVID (i.e., date of first COVID‐19 infection at least 3 months prior to the questionnaire date). Of the 200 people with long COVID defined in this way, 35 (17.5%), 46 (23.0%), 62 (31.0%) and 57 (28.5%) reported the date of first COVID or onset of symptoms to be 3–6 months, 6–12 months, 1–2 years and >2 years prior to the questionnaire date, respectively. In adjusted analyses, factors associated with having long COVID among those with a history of COVID‐19 were female gender compared to male, being unemployed or not working due to sickness/disability, not having money for basic needs, earlier HIV diagnosis date, having had a diagnosis of diabetes, asthma or COPD, having self‐reported BMI ≥30 kg/m^2^ and symptoms of depression and anxiety (Table 2).

**TABLE 2:** Factors associated with long COVID‐19 (ongoing COVID‐19 symptoms reported at least 3 months after the date of first positive test) in people with HIV with a history of COVID‐19.

*N* = 1844^a^		Long COVID (*N* = 200/1844)			
	Number with long COVID/history of COVID‐19 (%)	Unadjusted odds ratio (95% CI)	*p*‐value	Adjusted^b^ odds ratio (95% CI)	*p*‐value
Age group (years)					
18–34	12/183 (6.6%)	0.58 (0.30–1.13)	0.10^c^	0.60 (0.30–1.21)	0.70^c^
35–44	46/429 (10.7%)	1		1	
45–54	56/537 (10.4%)	0.97 (0.64–1.46)		0.83 (0.54–1.28)	
55–64	69/520 (13.3%)	1.27 (0.86–1.89)		1.04 (0.67–1.61)	
65+	17/175 (9.7%)	0.90 (0.50–1.61)		0.73 (0.39–1.36)	
Demographic group					
GBMSM	117/1218 (9.6%)	1	0.082	1	0.062
Black African heterosexual men	7/88 (8.0%)	0.81 (0.37–1.80)		0.78 (0.35–1.74)	
Other heterosexual men	16/129 (12.4%)	1.33 (0.76–2.33)		1.37 (0.78–2.42)	
Black African women	24/190 (12.6%)	1.36 (0.85–2.17)		1.27 (0.78–2.05)	
Other women	32/188 (17.0%)	1.93 (1.26–2.95)		1.92 (1.24–2.95)	
Non‐binary, other or undisclosed gender or sexuality^d^	4/31 (12.9%)	1.39 (0.48–4.05)		1.45 (0.49–4.24)	
Ethnicity					
White	137/1300 (10.5%)	1	0.45	1	0.35
Black African	32/295 (10.8%)	1.03 (0.69–1.55)		0.73 (0.45–1.18)	
Black other	9/40 (22.5%)	2.46 (1.15–5.29)		1.93 (0.87–4.28)	
Asian	8/74 (10.8%)	1.03 (0.48–2.19)		1.03 (0.48–2.23)	
Mixed	5/53 (9.4%)	0.88 (0.35–2.26)		0.82 (0.32–2.11)	
Other including undisclosed	9/82 (11.0%)	1.05 (0.51–2.14)		1.07 (0.52–2.23)	
Gender					
Male	142/1450 (9.8%)	1	0.016	1	0.029
Female	56/378 (14.8%)	1.60 (1.15–2.23)		1.71 (1.15–2.54)	
Non‐binary, other or undisclosed gender^d^	2/16 (12.5%)	1.32 (0.30–5.85)		1.44 (0.32–6.56)	
Region of HIV care					
London	91/919 (9.9%)	1	0.35	1	0.26
Midlands and East	37/298 (12.4%)	1.29 (0.86–1.94)		1.33 (0.88–2.03)	
North	33/282 (11.7%)	1.21 (0.79–1.84)		1.25 (0.81–1.93)	
South	34/323 (10.5%)	1.07 (0.71–1.62)		1.10 (0.71–1.68)	
Scotland/Wales/unknown	5/22 (22.7%)	2.68 (0.96–7.42)		2.77 (0.97–7.87)	
Highest education level					
Education to age 16	44/359 (12.3%)	1	0.21	1	0.31
Education to age 18 or post‐16 qualification	58/451 (12.9%)	1.06 (0.69–1.61)		1.14 (0.75–1.75)	
Undergraduate degree	49/471 (10.4%)	0.83 (0.54–1.28)		0.90 (0.58–1.39)	
Postgraduate degree	42/499 (8.4%)	0.66 (0.42–1.03)		0.72 (0.46–1.14)	
Other/missing	7/64 (10.9%)	0.88 (0.38–2.05)		0.83 (0.35–1.97)	
Employment status					
Employed	127/1392 (9.1%)	1	<0.0001	1	<0.0001
Unemployed	20/91 (22.0%)	2.80 (1.65–4.76)		2.61 (1.51–4.51)	
Retired	12/181 (6.6%)	0.71 (0.38–1.31)		0.57 (0.27–1.18)	
Not working due to sickness/disability	35/112 (31.3%)	4.53 (2.91–7.02)		3.92 (2.47–6.25)	
Other including student, carer	2/41 (4.9%)	0.51 (0.12–2.14)		0.48 (0.11–2.04)	
Having money for basic needs					
Yes, always	74/1101 (6.7%)	1	<0.0001^c^	1	<0.0001^c^
Most of the time	56/417 (13.4%)	2.15 (1.49–3.11)		2.29 (1.57–3.34)	
Some of the time	39/211 (18.5%)	3.15 (2.07–4.79)		3.62 (2.29–5.72)	
No	26/84 (31.0%)	6.22 (3.70–10.46)		7.83 (4.46–13.74)	
Household composition					
Living alone	65/569 (11.4%)	1	0.51^c^	1	0.49^c^
2 people	87/810 (10.7%)	0.93 (0.66–1.31)		0.93 (0.66–1.32)	
3–5 people	42/407 (10.3%)	0.89 (0.59–1.35)		0.84 (0.54–1.31)	
6 people or more	2/24 (8.3%)	0.70 (0.16–3.07)		0.88 (0.20–3.90)	
Year of HIV diagnosis					
2014–2023	37/453 (8.2%)	1	0.001^c^	1	0.007^c^
2008–2013	45/524 (8.6%)	1.06 (0.67–1.66)		0.92 (0.57–1.48)	
2002–2007	58/442 (13.1%)	1.70 (1.10–2.63)		1.50 (0.93–2.42)	
1996–2001	35/250 (14.0%)	1.83 (1.12–2.99)		1.58 (0.92–2.72)	
1995 or earlier	25/175 (14.3%)	1.88 (1.09–3.22)		1.75 (0.96–3.19)	
Most recent viral load (self‐report)					
<50 copies/mL	183/1748 (10.5%)	1	0.33	1	0.47
≥50 copies/mL	7/47 (14.9%)	1.50 (0.66–3.39)		1.36 (0.59–3.12)	
Don't know/missing	10/49 (20.4%)	2.19 (1.08–4.47)		2.01 (0.97–4.19)	
Cardiovascular disease					
No	159/1594 (10.0%)	1		1	
Ever‐diagnosed	18/112 (16.1%)	1.73 (1.02–2.94)	0.043	1.57 (0.90–2.73)	0.11
Diabetes					
No	147/1565 (9.4%)	1		1	<0.0001
Ever‐diagnosed	28/135 (20.7%)	2.52 (1.61–3.96)	<0.0001	2.43 (1.50–3.93)	
Asthma or COPD					
No	133/1434 (9.3%)	1		1	
Ever‐diagnosed	49/306 (16.0%)	1.87 (1.31–2.66)	0.001	1.85 (1.28–2.65)	0.001
BMI (self‐reported)					
<30 kg/m^2^	127/1333 (9.5%)	1		1	
Obese (≥30 kg/m^2^)	59/380 (15.5%)	1.75 (1.25–2.43)	0.001	1.62 (1.13–2.32)	0.009
Depressive symptoms					
No	119/1497 (7.9%)	1		1	
Yes (PHQ‐9 ≥10)	78/324 (24.1%)	3.67 (2.68–5.04)	<0.0001	3.80 (2.74–5.28)	<0.0001
Anxiety symptoms					
No	142/1585 (9.0%)	1		1	
Yes (GAD‐7 ≥10)	56/237 (23.6%)	3.14 (2.23–4.44)	<0.0001	3.12 (2.18–4.46)	<0.0001
Number of sexual partners in the last 3 months					
0	71/480 (14.8%)	1	0.023^c^	1	0.11^c^
1	52/625 (8.3%)	0.52 (0.36–0.76)		0.56 (0.38–0.84)	
2–5	23/316 (7.3%)	0.45 (0.28–0.74)		0.52 (0.31–0.88)	
>6	25/228 (11.0%)	0.71 (0.44–1.15)		0.83 (0.49–1.39)	
Number of COVID vaccine doses					
Sufficient (3 or 4)	164/1560 (10.5%)	1		1	
Insufficient (0, 1 or 2)	34/268 (12.7%)	1.24 (0.83–1.83)	0.29	1.14 (0.75–1.74)	0.54

*Note*: Region: Midlands and East of England (East Midlands, East of England, West Midlands); North of England (North East, North West, Yorkshire and Humber); South of England (South East, South West). Missing categories if not incorporated into one of the above categories are included in analyses but not shown in the table.

Abbreviations: BMI, body mass index; COPD, chronic obstructive pulmonary disease; GBMSM, gay, bisexual and other men‐who‐have‐sex‐with‐men.

^a^ Participant included in analysis reported a previous positive COVID‐19 test and had a date when they first had COVID‐19. Number of missing values were not included in categories specified in table: employment status (*n* = 27), money for basic needs (*n* = 31), household composition (*n* = 34), cardiovascular disease (*n* = 138), diabetes (*n* = 144), asthma or COPD (*n* = 104), self‐reported BMI/obesity (*n* = 131), depressive symptoms (*n* = 23), anxiety symptoms (*n* = 22), number of sexual partners in the last 3 months (*n* = 195), number of COVID vaccine doses (*n* = 16).

^b^ Every factor of interest was considered in a separate model for all results. Multivariable models adjusted for age group, ethnicity, gender and year of HIV diagnosis.

^c^ *p*‐value refers to test for trend.

^d^ Combined group of non‐binary, other gender and undisclosed gender or sexuality because of small group size for statistical analyses.

### COVID‐19 vaccine uptake and predictors of incomplete vaccine uptake

Overall, 4121 participants completed the question regarding the number of COVID‐19 vaccines received. Of these, 95.8% (3946) had received at least one dose, indicating that levels of COVID‐19 vaccine uptake were very high. Uptake of at least one dose of vaccine by ethnicity was 96.4%, 94.8%, 86.6%, and 98.8% among White, Black African, Black other, and Asian ethnicity respectively; by gender was 96.5% in men and 94.0% in women; and by age was 93.2%, 93.2%, 95.8%, 96.8%, and 98.3% among 18–34, 35–44, 45–54, 55–64, and over 65‐year‐olds respectively. Of those that had had at least one dose (*n* = 3946), 0.8% (*n* = 32) received one dose only; 11.2% (*n* = 442) received two doses, 46.6% (*n* = 1840) received three doses, and 41.4% (*n* = 1632) had received four or more doses.

**FIGURE 2:**
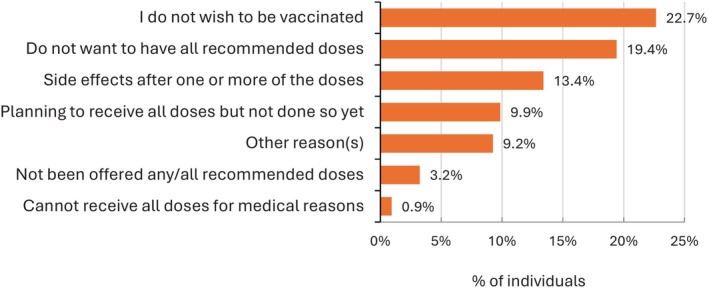
Reasons given for not having received all the recommended doses of SARS‐CoV‐2 vaccine (*n* = 649).

Overall, 84.3% (3472/4121) of participants reported sufficient uptake of COVID‐19 vaccination (defined as at least three vaccine doses for people with HIV), and 649 (15.7%, 95% CI: 14.6%‐16.9%) had not (Table 3). The most common reasons given for incomplete vaccine uptake were not wishing to be vaccinated (147/649, 22.7%), not wanting to have all recommended doses (126/649, 19.4%) (Figure 2) and experiencing vaccine side effects after one or more doses (87/649, 13.4%). Only 0.9% of participants declared a medical contraindication to the vaccine.

**TABLE 3:** Factors associated with incomplete COVID‐19 vaccine uptake (less than three doses) among people with HIV.

		Incomplete COVID‐19 vaccine uptake (*N* = 649/4121)			
*N* = 4121^a^	Number with incomplete vaccine uptake / number of participants (%)	Unadjusted odds ratio (95% CI)	*p*‐value	Adjusted^b^ odds ratio (95% CI)	*p*‐value
Age group (years)					
18–34	95/310 (30.6%)	1.60 (1.19–2.14)	<0.0001^c^	1.60 (1.17–2.19)	<0.0001^c^
35–44	173/798 (21.7%)	1		1	
45–54	194/1220 (15.9%)	0.68 (0.54–0.86)		0.65 (0.51–0.83)	
55–64	146/1262 (11.6%)	0.47 (0.37–0.60)		0.51 (0.39–0.66)	
65+	41/531 (7.7%)	0.30 (0.22–0.43)		0.39 (0.27–0.57)	
Demographic group					
GBMSM	232/2449 (9.5%)	1	<0.0001	1	<0.0001
Black African heterosexual men	64/250 (25.6%)	3.29 (2.40–4.50)		3.74 (2.70–5.19)	
Other heterosexual men	65/346 (18.8%)	2.21 (1.63–2.99)		2.83 (2.07–3.88)	
Black African women	149/543 (27.4%)	3.61 (2.87–4.56)		3.61 (2.83–4.61)	
Other women	116/435 (26.7%)	3.47 (2.70–4.47)		3.67 (2.83–4.75)	
Non‐binary, other or undisclosed gender or sexuality^d^	23/98 (23.5%)	2.93 (1.80–4.77)		2.88 (1.75–4.75)	
Ethnicity					
White	311/2722 (11.4%)	1	<0.0001	1	<0.0001
Black African	225/848 (26.5%)	2.80 (2.31–3.40)		1.88 (1.48–2.38)	
Black other	41/112 (36.6%)	4.48 (2.99–6.69)		3.62 (2.38–5.51)	
Asian	18/161 (11.2%)	0.98 (0.59–1.62)		0.72 (0.43–1.21)	
Mixed	22/124 (17.7%)	1.67 (1.04–2.69)		1.30 (0.79–2.12)	
Other including undisclosed	32/154 (20.8%)	2.03 (1.35–3.05)		1.53 (1.00–2.34)	
Gender					
Male	372/3089 (12.0%)	1	<0.0001	1	<0.0001
Female	265/978 (27.1%)	2.71 (2.27–3.24)		1.95 (1.57–2.42)	
Non‐binary, other or undisclosed gender^d^	12/54 (22.2%)	2.09 (1.09–4.00)		1.61 (0.82–3.15)	
Region of HIV care					
London	328/1955 (16.8%)	1	0.0001	1	0.0030
Midlands and East	115/696 (16.5%)	0.98 (0.78–1.24)		0.84 (0.66–1.08)	
North	119/651 (18.3%)	1.11 (0.88–1.40)		1.12 (0.88–1.43)	
South	83/774 (10.7%)	0.60 (0.46–0.77)		0.65 (0.50–0.85)	
Scotland/Wales/unknown	4/45 (8.9%)	0.48 (0.17–1.36)		0.44 (0.15–1.27)	
Highest education level					
Education to age 16	159/965 (16.5%)	1	0.0001	1	0.0001
Education to age 18 or post‐16 qualification	178/1068 (16.7%)	1.01 (0.80–1.28)		1.01 (0.79–1.29)	
Undergraduate degree	149/937 (15.9%)	0.96 (0.75–1.22)		0.84 (0.65–1.09)	
Postgraduate degree	114/965 (11.8%)	0.68 (0.52–0.88)		0.62 (0.47–0.81)	
Other/missing	49/186 (26.3%)	1.81 (1.25–2.62)		1.41 (0.95–2.10)	
Employment status					
Employed	406/2700 (15.0%)	1	<0.0001	1	<0.0001
Unemployed	91/304 (29.9%)	2.41 (1.85–3.15)		2.18 (1.64–2.89)	
Retired	39/553 (7.1%)	0.43 (0.30–0.60)		0.95 (0.61–1.47)	
Not working due to sickness/disability	60/359 (16.7%)	1.13 (0.84–1.53)		1.58 (1.15–2.16)	
Other including student, carer	35/129 (27.1%)	2.10 (1.41–3.15)		1.47 (0.96–2.25)	
Having money for basic needs					
Yes, always	229/2178 (10.5%)	1	<0.0001^c^	1	<0.0001^c^
Most of the time	178/1062 (16.7%)	1.71 (1.39–2.12)		1.43 (1.15–1.79)	
Some of the time	137/531 (25.8%)	2.96 (2.33–3.75)		1.89 (1.46–2.46)	
No	81/271 (29.9%)	3.63 (2.70–4.87)		2.57 (1.88–3.53)	
Household composition					
Living alone	185/1408 (13.1%)	1	<0.0001^c^	1	0.010^c^
2 people	210/1710 (12.3%)	0.93 (0.75–1.14)		0.81 (0.65–1.01)	
3–5 people	207/853 (24.3%)	2.12 (1.70–2.64)		1.21 (0.95–1.54)	
6 people or more	26/62 (41.9%)	4.77 (2.82–8.09)		2.69 (1.54–4.69)	
Year of HIV diagnosis					
2014–2023	182/915 (19.9%)	1	<0.0001^c^	1	0.021^c^
2008–2013	162/1078 (15.0%)	0.71 (0.56–0.90)		0.71 (0.55–0.91)	
2002–2007	198/1087 (18.2%)	0.90 (0.72–1.12)		0.92 (0.71–1.19)	
1996–2001	72/604 (11.9%)	0.55 (0.41–0.73)		0.69 (0.50–0.95)	
1995 or earlier	35/437 (8.0%)	0.35 (0.24–0.51)		0.61 (0.40–0.91)	
Last plasma HIV‐RNA					
<50 cp/mL	563/3834 (14.7%)	1	<0.0001	1	<0.0001
≥50 cp/mL	36/139 (25.9%)	2.03 (1.38–3.00)		1.87 (1.24–2.81)	
Don't know/missing	50/148 (33.8%)	2.96 (2.08–4.22)		2.52 (1.73–3.68)	
Cardiovascular disease					
No	535/3379 (15.8%)	1	0.13	1	0.091
Ever‐diagnosed	44/346 (12.7%)	0.77 (0.56–1.08)		1.36 (0.95–1.94)	
Diabetes					
No	538/3358 (16.0%)	1	0.16	1	0.43
Ever‐diagnosed	45/343 (13.1%)	0.79 (0.57–1.10)		0.87 (0.61–1.23)	
Asthma or COPD					
No	492/3098 (15.9%)	1	0.12	1	0.23
Ever‐diagnosed	95/701 (13.6%)	0.83 (0.66–1.05)		0.86 (0.67–1.10)	
BMI (self‐reported)					
<30 kg/m^2^	385/2815 (13.7%)	1		1	
Obese (≥30 kg/m^2^)	158/886 (17.8%)	1.37 (1.12–1.68)	0.002	0.95 (0.76–1.19)	0.64
Depressive symptoms					
No	449/3213 (14.0%)	1	<0.0001	1	<0.0001
Yes (PHQ‐9 ≥10)	179/831 (21.5%)	1.69 (1.39–2.05)		1.69 (1.38–2.07)	
Anxiety symptoms					
No	500/3439 (14.5%)	1	0.0001	1	<0.0001
Yes (GAD‐7 ≥10)	126/602 (20.9%)	1.56 (1.25–1.94)		1.51 (1.20–1.90)	
Number of sexual partners in the last 3 months					
0	191/1239 (15.4%)	1	0.012^c^	1	0.06^c^
1	225/1309 (17.2%)	1.14 (0.92–1.41)		0.85 (0.67–1.06)	
2–5	63/578 (10.9%)	0.67 (0.50–0.91)		0.68 (0.49–0.95)	
>6	45/377 (11.9%)	0.74 (0.53–1.05)		0.76 (0.52–1.10)	
Ever positive COVID‐19 test					
No	333/2071 (16.1%)	1		1	
Yes	316/2050 (15.4%)	0.95 (0.80–1.12)	0.55	0.92 (0.77–1.10)	0.35

*Note*: Region: Midlands and East of England (East Midlands, East of England, West Midlands); North of England (North East, North West, Yorkshire and Humber); South of England (South East, South West). Missing categories if not incorporated into one of the above categories are included in analysis but not shown in the table.

Abbreviations: BMI, body mass index; COPD, chronic obstructive pulmonary disease; GBMSM, gay, bisexual and other men‐who‐have‐sex‐with‐men.

^a^ 649 people had incomplete vaccination (less than three doses) out of 4121 who completed the question regarding number of COVID‐19 vaccines received. Number of missing values were not included in categories specified in table: employment status (*n* = 76), money for basic needs (*n* = 79), household composition (*n* = 88), cardiovascular disease (*n* = 396), diabetes (*n* = 420), asthma or COPD (*n* = 322), self‐reported BMI/obesity (*n* = 420), depressive symptoms (*n* = 77), anxiety symptoms (*n* = 80), number of sexual partners in the last 3 months (*n* = 618).

^b^ Every factor of interest was considered in a separate model for all results. Multivariable models adjusted for age group, ethnicity, gender, and year of HIV diagnosis.

^c^ *p*‐value refers to test for trend.

^d^ Combined group of non‐binary, other gender and undisclosed gender or sexuality because of small group size for statistical analyses.

In terms of demographic factors, incomplete COVID‐19 vaccine uptake was more likely among women, participants who were younger and those of Black ethnicity. For the demographic group classification, incomplete vaccine uptake was more likely among all other groups compared to GBMSM (Table 3). In adjusted analysis, demographic factors associated with incomplete vaccine uptake were younger age (aOR 1.60, 95% CI 1.17–2.19 for participants aged 18–34 compared with those aged 35–44), female gender (aOR 1.95, 95% CI 1.57–2.42 compared with men), Black ethnicity (aOR 1.88, 95% CI 1.48–2.38 for Black African ethnicity and aOR 3.62, 95% CI 2.38–5.51 for Black other ethnicity compared with White ethnicity) and demographic group. Indicators of poor socio‐economic status were also strongly associated with incomplete vaccine uptake: financial hardship (aOR 2.57, 95% CI 1.88–3.53 for not having money for basic needs compared with always having money for basic needs), non‐employment (aOR 2.18, 95% CI 1.64–2.89 for unemployment and aOR 1.58, 95% CI 1.15–2.16 for not working due to sickness/disability compared with those employed), lower education level (aOR 0.62, 95% CI 0.47–0.81 for postgraduate degree vs. education to age 16) and living in a household of six people or more (aOR 2.69, 95% CI 1.54–4.69 compared with people who lived alone). We also found that incomplete vaccine uptake was more likely among those in London and the Northern region compared to the Southern region, among those with a more recent HIV diagnosis, among those with self‐reported detectable or unknown viral load compared to undetectable, and among participants with symptoms of depression or anxiety.

## DISCUSSION

Positive Voices 2022 is the largest UK survey in people with HIV and took place directly following a highly disruptive episode for health and social care systems due to the COVID‐19 pandemic. Overall, the pandemic had profound impacts on general population health and wellbeing with a disproportionate effect on many who already faced disadvantage and financial hardship [30]. COVID‐19 and its wide‐ranging impacts also exacerbated health inequalities in people with HIV. In this study, the most commonly reported impacts of the pandemic on daily life among people with HIV were lack of social contact and adverse effects on mental health, access to healthcare, and financial security.

At the time of the survey, half of the participants reported having previously tested positive for COVID‐19. This was more common among participants who were younger, identified as GBMSM, were employed, had higher education levels, were financially secure, shared their housing with other people, and had more than one sexual partner in the last 3 months. Many of these factors are known to be associated with greater levels of social mixing, which increase the risk of being infected with COVID‐19 [31]. The presence of other comorbidities and of symptoms of depression or anxiety may reduce levels of social mixing, which would explain the lower prevalence of a history of COVID‐19 associated with these factors in this study. In particular, during the pandemic period, people with HIV who had other health conditions may have avoided or reduced social contact to reduce the risk of acquiring a COVID‐19 infection.

In this study, 11% (200/1844) of people with HIV who had a history of COVID‐19, reported ongoing symptoms of COVID‐19 at 12 weeks or more since the onset of COVID‐19, consistent with long COVID [32]. Symptoms of long COVID include fatigue, cognitive impairment, and autonomic dysfunction as well as other physical and psychological manifestations [32, 33], but the underlying pathogenesis and therapeutic options remain unclear [34]. The reported prevalence of long COVID varies widely. The UK's Office for National Statistics (ONS) reported in March 2023 that approximately 2.6% of the UK population reported long COVID symptoms 12 weeks or more after COVID‐19 infection [2]. However, the WHO has reported a prevalence of 10%–20%, and studies that used matched control groups (without previous COVID‐19 infection) estimate the prevalence of long COVID symptoms after 12 weeks in the general population of 12.6% in a Dutch study and 6.6% at 6 months in a study using Scottish data [34, 35], which is more consistent with our findings. There are clinical, socio‐demographic, and immunological factors that may place people with HIV at increased risk of long COVID. Some studies, though often based on small cohorts, suggested a higher incidence of long COVID in people with HIV compared to HIV‐negative individuals, but findings have varied [32, 33].

We found several socio‐demographic and health‐related factors were associated with reporting long COVID, including female gender, financial hardship, unemployment, earlier HIV diagnosis date, living with other comorbidities such as diabetes, asthma/COPD, being obese (BMI ≥30) and having symptoms of depression or anxiety. The association of long COVID with symptoms of anxiety and depression has been reported elsewhere [33, 34], though the cross‐sectional nature of our study makes it impossible to identify if symptoms of anxiety and depression predated or followed the development of long COVID. However, while there remains a lack of treatment options for long COVID, screening for and treating symptoms of anxiety and depression in people with HIV with long COVID may give symptomatic benefit. The association of increased risk of long COVID in those of female gender has also been previously reported [36]; associations between ethnicity and long COVID remain less clear currently [37].

Overall, we found 95.8% of participants in PV2022 had received at least one dose of a COVID‐19 vaccine, and this was high (>86%) across all ethnic groups. In the general UK adult population, by the end of August 2022, 93.6% had received at least one dose of a COVID‐19 vaccine and 88.2% also had a second dose, though this varied by ethnicity, geographical location, and socio‐economic circumstances [2]. In particular, uptake of one dose of a COVID‐19 vaccine in Black African individuals in the United Kingdom was 60%–65% and for people of Black Caribbean ethnicity even lower at 55%–60% [2] compared to over 90% in people of White ethnicity. Additionally, REACT‐2, which was a repeated cross‐sectional community survey of adults in England, also reported that vaccine confidence varied by ethnicity and was highest in those of White ethnicity at 92.6% and lowest in those of Black ethnicity at 72.5% [38]. Vaccine confidence also varied by age and sex in REACT‐2 (higher in older groups and lower in women). The most cited reasons in REACT‐2 for declining the vaccine were concerns about long‐term health effects or side effects, with additional free text comments highlighting concerns about pregnancy and future fertility.

Notably, PV2022 found a higher prevalence of receiving at least one dose of the COVID‐19 vaccine among people with HIV of Black African ethnicity (94.8%), compared to people of Black ethnicity in the UK general population (65%). This may be linked to higher levels of engagement with healthcare services among people living with HIV, as well as high levels of trust and satisfaction with HIV services: PV2022 participants gave a mean HIV clinic satisfaction rating of 9.4 out of 10, with very high satisfaction across a range of patient‐reported experience measures [39]. This level of trust and engagement in HIV care may have facilitated the dissemination of information about the benefits of COVID‐19 vaccination and helped address any misinformation people may have received. However, it may also be an indication that people with HIV considered themselves at higher risk of severe outcomes from COVID‐19 as they were prioritized for earlier vaccination schedules [21] than national prioritization based on age criteria.

While single doses of COVID‐19 vaccines offered some protection, receiving the full recommended course of COVID‐19 vaccination, including boosters, substantially reduced the risk of severe disease and hospitalization in the general population and in people with HIV [40]. In PV2022, we found an incomplete vaccine course uptake (less than three vaccine doses) was reported by 16% of participants. Compared to GBMSM, incomplete vaccine uptake was more likely in all other demographic groups, including Black African heterosexual men, other heterosexual men, Black African women and other women. Younger age, lower educational attainment, unemployment, financial insecurity, living with at least six others, more recent HIV diagnosis, having detectable or unknown viral load, and symptoms of depression and anxiety were also associated with incomplete vaccine uptake, although the diagnosed comorbidities were not. It is possible that lower uptake of the complete vaccine course in women in our study may be partly related to inconsistent early messaging and misinformation around vaccine use and pregnancy and/or fertility. A recent meta‐analysis [22] of factors associated with COVID‐19 vaccine acceptance in people with HIV reported similar results to ours, that men had a higher likelihood of vaccine acceptance than women (odds ratio (OR), 2.06; 95% CI, 1.16–3.66); people with HIV aged less than 40 years had significantly lower odds of acceptance than those aged 40 years and above (OR, 0.70; 95% CI, 0.54–0.90); and those with a lower level of education had a lower likelihood of acceptance (OR, 0.60; 95% CI, 0.40–0.89). The review also found that participants' vaccine‐related perceptions and attitudes, including concerns about vaccine efficacy and safety or fear of contracting COVID‐19, were significantly negatively predictive of vaccine acceptability and uptake.

## LIMITATIONS

Although largely representative of people with HIV currently in care in the United Kingdom, PV2022 slightly underrepresented people of Black African ethnicity, younger adults and heterosexual individuals compared with the overall UK population with HIV [39]. Those who participated in the survey and completed the sections on COVID‐19 may differ from those who did not with respect to socio‐demographic and health factors. Data on COVID‐19 infection and on vaccine uptake were self‐reported rather than linked to laboratory and immunization records. We also did not collect information on multiple episodes of COVID‐19. In addition, limited data were collected on persistent symptoms following COVID‐19 infection in order to meet the basic definition for long COVID, but more detail such as duration, severity and specific symptoms was not collected. The cross‐sectional survey design means causal inference is not possible, and the data are susceptible to recall bias, which may limit interpretation of results. Furthermore, we did not collect information on biomedical factors that may have influenced the development of post‐COVID syndrome, so we could not investigate factors such as SARS‐CoV‐2 persistence, neuroinflammation, abnormal clotting and immune dysfunction. Finally, the quantitative nature of the study prevented us from fully capturing the diversity of barriers and facilitators to the uptake of vaccination.

## CONCLUSIONS

Our results indicate a that a significant proportion of people living with HIV felt the COVID‐19 pandemic had a negative impact on their social contact, mental health, healthcare access and financial security, which may suggest a widening of existing inequality among people with HIV. We found 11% of those who had previously tested positive for COVID‐19 had ongoing symptoms more than 3 months after COVID‐19 onset (long COVID) which was associated with several key factors including female gender, socio‐economic disadvantage, living with other comorbidities such as diabetes, lung disease and obesity and having poor mental health. In the United Kingdom, cultural contexts impacted levels of COVID‐19 vaccine uptake in the general population. The high uptake of at least one dose of the COVID‐19 vaccine across all ethnic groups in PV2022 compared to the UK general population might reflect the important role of HIV healthcare staff as a trusted source of information to address vaccine concerns, build vaccine confidence and combat the proliferation of misleading and false information, particularly through social media, termed an ‘infodemic’ by WHO [22]. However, we also documented incomplete COVID‐19 vaccine uptake of all recommended doses in 16% of participants, with lower uptake in women, young people, Black African individuals, those with socio‐economic disadvantage and those with symptoms of poor mental health. This suggests that there was a need for appropriately targeted vaccine uptake interventions among key population groups.

## AUTHOR CONTRIBUTIONS

*Study concept and design*: M. Kall, C. Smith, C. Kelly, F. C. Lampe and A. J. Rodger. *Statistical analysis*: F. Nakagawa and R. Palich. *Acquisition of data*: M. Kall, J. Sewell, C. Kelly, A. Aghaizu, N. Mackie, F. C. Lampe and A. J. Rodger. *Interpretation of data*: F. Nakagawa, R. Palich, M. Kall, J. Sewell, C. Smith, A. Pelchen‐Matthews, A. Aghaizu, A. Sparrowhawk, C. Humphreys, F. C. Lampe and A. J. Rodger. *Drafting of the manuscript*: F. Nakagawa, R. Palich and A. J. Rodger. *Critical revision of the manuscript for important intellectual content*: All authors. *Full access to all the data and responsibility for its integrity and the data analysis*: F. Nakagawa.

## FUNDING INFORMATION

This study represents independent research funded by the National Institute for Health Research (Programme Grants for Applied Research, A person‐centred Needs Informed model of Care for people with HIV, to improve wellbeing, mental health and reduce socio‐economic disadvantages and stigma, [NICHE]: NIHR202038). The views expressed in this publication are those of the authors and not necessarily those of the NHS, the National Institute for Health Research or the Department of Health and Social Care. Funding was also received from Gilead Sciences Ltd.

## CONFLICT OF INTEREST STATEMENT

The authors declare no conflicts of interest.

## Supporting information


**Data S1.** Supporting information.
